# Enhancing collaborative learning in health management education: an investigation of Padlet-mediated interventions and the influence of flexible thinking

**DOI:** 10.1186/s12909-023-04796-y

**Published:** 2023-11-08

**Authors:** Lior Naamati-Schneider, Dorit Alt

**Affiliations:** 1 Health Systems Management Department, Hadassah Academic College, Jerusalem, Israel; 2 Faculty of Education and Instruction, Tel Hai College, Upper Galilee, Israel

**Keywords:** Online collaborative learning, Padlet, Health management, Higher education

## Abstract

This study had three primary goals. First, it aimed to craft an intervention program centered around collaborative learning enabled by Padlet. Second, it aimed to gauge the perceptions of health management students regarding this intervention and how it affected their collaborative learning experiences. Additionally, the third objective of the study aimed to investigate how students’ flexible thinking within the learning process might shape their perceptions of the advantages derived from this instructional activity within the domain of online collaborative learning. Data for the analysis were gathered from 100 Israeli undergraduate students by two measurements: *Flexible thinking in learning and Student perceptions of collaborative learning via Padlet*. The intervention program included several stages. First, the students discussed the pedagogic objective of using Padlet. In the second stage, the students were presented with ill-structured problems related to the course content. Each group had to choose one problem and analyze it from three perspectives discussed in the course—healthcare provider, patient, and organization. Next, the students presented and explained their solutions employing the shared knowledge base. The final work was presented in different formats using various technologies. The PLS-SEM analysis has corroborated our hypothesis that students’ flexible thinking might positively contribute to their perception of Padlet utilization. According to the empirical model, in general, students who perceived themselves as more flexible were found more receptive to utilizing the proposed technological tool (Padlet) and hence tended to appreciate its function as a collaborative learning platform enabler. This study mainly underscores the important role flexible thinking plays in motivating managers and medical professionals to embrace innovative technologies or methods for teamwork, that could enable them to weigh arguments, seek alternative solutions to authentic problems, and adjust their approaches effectively and collaboratively as new challenges emerge.

## Introduction

Padlet is a web-based tool for online collaborative learning (OCL), resembling a bulletin board where users can share multimedia content [1, 2]. Recent studies have highlighted the advantages of Padlet-mediated learning in enhancing student engagement and facilitating positive collaborative learning experiences [3–6]. Additionally, other research [1, 7] has shown that Padlet technology promotes user engagement and cooperation. However, despite its proven effectiveness, Padlet remains underutilized in higher education, particularly within healthcare professions. To bridge this gap, our research pursued three core aims. We sought to design an intervention program that leverages Padlet for student collaboration and assess how health management students perceived this intervention’s impact on collaborative learning.

This study also delved into the influence of students’ flexible thinking on their inclination to embrace Padlet-based learning and recognize its benefits for OCL. Flexible thinking is vital in online learning due to increased autonomous interactions. It involves adaptability, openness to different views, and balancing various needs to optimize outcomes [8, 9].

However, the pivotal role of flexible thinking in learning has received relatively limited attention in the context of healthcare disciplines and online collaborative learning through Padlet, prompting the need for this investigation.

Thus, this study explored the utilization of a Padlet-mediated online tool as a vehicle for collaborative learning among undergraduate health management students. It aimed to evaluate how the adoption of technology-enhanced collaborative practices intersected with students’ cognitive flexibility [10]. This study’s results can enhance our understanding of tech-supported OCL, especially using lesser-known tools like Padlet, in achieving educational goals and promoting higher-order learning among undergraduates. A thorough understanding of the factors shaping students’ perspectives on technology integration in their courses and OCL is essential for academic institutions. This is key to navigating the changing healthcare landscape shaped by demographics, technology, and politics. With the surge in medical demands and technology, global health systems must adopt digital innovations like AI and telemedicine. Modern challenges require new skills, underscoring the urgency for health and academic entities to adapt [11–13]. This research not only fills a critical research gap but also underscores its significance in shaping the future of healthcare education and online collaborative learning practices.

### Literature review

In this section, we will review the advantages as well as challenges of collaborative and online learning. We will also address Padlet-mediated learning and flexible thinking in technology-enhanced environments by reviewing relevant recent literature. Further, we will present the use of this teaching methodology in the healthcare professions and examine how it impacts the skills required of today’s healthcare students.

### Online collaborative learning

Collaborative learning is a growing pedagogic approach where learners work with peers, engaging in activities like questioning, discussion, brainstorming, reflection, and decision-making to promote active learning [14]. Collaborative learning has been shown to improve performance in a variety of fields by making learners actively engaged in learning and increasing their satisfaction, motivation, and well-being [15].

This form of learning was found to be effective for coping with the challenges of active teaching, especially in big courses where only a handful of students are active participants [16]. Collaborative learning fosters a supportive environment, ensuring equal opportunity and encouraging participation through small group work [1]. This emerging pedagogy turns learning from an individual activity into a collaborative process, offering benefits like enhanced knowledge acquisition, self-regulation, positive interdependence, and increased engagement [1, 17, 18].

In recent years, with the improved performance and increased accessibility of digital technologies, many academic institutions have begun to assimilate OCL into their courses [14, 19]. The constraints of social distancing imposed by the Covid 19 pandemic have enhanced that trend. OCL is defined as a goal-oriented activity of a group of students that are committed to achieving a shared target and creating new knowledge by interactively learning in a digital environment [20]. Numerous studies have indicated the advantages of OCL [21–23]. These benefits encompass enhanced study motivation, better learner communication, fostering social activism, cultivating advanced thinking skills, and being inclusive for students from varied backgrounds. As learners engage with peers, they brainstorm, assess suggestions, gain feedback, and experience peer tutoring [24].

Social interaction in a learning environment includes interactions with peers (classmates or team members), interaction with the instructor, and academic involvement. Such interactions are considered central to achieving learning goals and improving academic performance in OCL [25–28]. Research has indicated that online learning technologies and distant learning increase learners’ motivation because the material becomes accessible anytime and from anywhere [29, 30]. Furthermore, online learning technologies invite constant interaction with instructors and peers, thus enhancing internet-based interpersonal relationships with fellow learners, which leads to better learning outcomes [31].

In CL, outcomes progress through stages. Initially, there’s idea-generating (IG) involving brainstorming and discussions, introducing learners to varied viewpoints [32, 33]. Next is idea organizing (IO), where initial ideas are analyzed and synthesized by the group [34]. Here, the instructor guides learners on tools and encourages higher-order learning [35]. The subsequent phase, intellectual convergence (IC), focuses on information processing, including reflections, discussions, and critical thinking, fostering a collaborative learning environment [35, 36]. Due to the advantages specified above and to meet the demands of the 21^st^ century and deal with the limitations of face-to-face education, OCL has become central in the evolution of academia as it adjusts to the changing reality [37–39].

### Online collaborative learning in health management education

Global changes and easy information access have reshaped the health industry, prompting new challenges for health workers. This demands new behaviors from both healthcare providers and administrations [12, 40]. Training for health administrators must now prioritize competency-based learning, emphasizing the development of capacities such as cognitive and interpersonal skills. Given the substantial interactions within ever-shifting health systems, suitable training can enhance the industry’s capacity to navigate current dynamic demands [41, 42]. Recently, cognitive skills like digital literacy and critical thinking, along with personal and interpersonal skills like communication, flexibility, creativity and teamwork, have become vital for shaping administrations that can meet the needs of 21^st^-century health systems [13].

OCL research in the health professions, including health administration, has shown that digital environments improve the training of students through collaborative learning and problem-solving, enabling students to acquire knowledge and higher-order thinking [43]. The health industry’s practical nature necessitates skills like flexibility and decision-making due to diverse team compositions [44]. Early exposure to collaborative online learning environments is essential for honing these skills during professional training [45]. It’s widely agreed that adopting these pedagogies across all learning levels will enable the health system to meet market demands, benefiting both the system and its patients in the coming decades [46].

### Online collaborative learning mediated by Padlet

Padlet, an OCL platform, functions as a virtual bulletin board for various multimedia posts and is always accessible to learners [1, 2]. Research shows its value in enhancing student engagement and positive collaborative learning experiences [3–6]. Further, studies like Gasmi and Thomas [7] emphasize its role in fostering user cooperation. For example, Padlet was found to enhance engagement among health and science students, fostering collaborative learning. Its use improved learning efficiency and provided a comfortable platform for communication and teamwork. Similarly, Garnham and Betts [47] detected a rise in students’ engagement in seminar courses when Padlet was used; In Beltrán-Martín’s study [48], students expressed satisfaction and improved academic performance using Padlet. Its advantages include ease of use, long-term content accessibility, and access to diverse resources. Given these features, Padlet supports a holistic, student-centered approach, allowing students to engage using their preferred medium and create content [1].

### Flexible thinking in health management studies

Spiro and Jehng [49] have initially defined cognitive flexibility as “the ability to adaptively re-assemble diverse elements of knowledge to fit the particular needs of a given understanding or problem-solving situation”. More recently, Tseng et al. [50] defined flexible thinking as “a person’s awareness of interaction and solution alternatives, ability to adapt to new situations, willingness to consider different opinions, and self-efficacy in being flexible” (p. 2289). Cognitive flexibility, encompassing open-mindedness and adaptability, is key to handling challenges and thriving in dynamic learning environments. Spiro and Jehng emphasize its importance for learning and problem-solving. It’s essential for online learning with autonomous interactions, making it a pivotal 21st-century skill [9]. Cognitive flexibility is a continuous process involving interactions with one’s environment, consciousness, embracing new perspectives, and adaptive behavior for improved results [8]. It’s considered higher-order thinking and part of executive functions [51]. It allows reevaluation of problems from unique angles [52] and aids students in understanding varied opinions, leading to solutions in diverse settings [53].

Cognitive flexibility positively influences teamwork [54]. In academia, it aids learners in adapting to new content and problem-solving [55, 56]. In medical fields, this skill is crucial, especially in therapeutic and managerial roles, making it vital for health professions [44]. Ernawati and Bratajaya [57] emphasized the importance of flexibility for nurses due to their interactions with patients and families. Flexible thinking enhances resilience in healthcare professionals [54] and is crucial in training mental health experts and adaptive therapies [58].

### Flexible thinking in technology-enhanced learning

Successful OCL implementation relies on factors like suitable technology, peer collaboration, and social engagement, which influence how learners and teachers adopt these technologies [59]. Another central precursor shown in previous research is student flexible thinking. Flexible thinking in learning is comprised of three main factors, which lie at the core of the model used for this research [10]. The first factor is technology acceptance. As technology integrates into education, students must show increased adaptability and cognitive flexibility [60]. The second factor, considered an integral part of cognitive flexibility, is open-mindedness. The ability to weigh various options and opinions and consider alternative solutions [61]. Individuals or teams with this ability are open to new ideas and capable of processing new knowledge when coping with evolving realities [55]. Open-mindedness is vital in education. Learners often rely on basic generalizations, which aren’t suitable for complex environments. To foster cognitive flexibility, students should be challenged with intricate problems and exposed to diverse opinions and views [62]. A key component of cognitive flexibility is adaptability to new situations, which involves one’s ability or motivation to adjust to changing contexts. In today’s education, rapid adaptability to novel methods and learning environments is crucial [63].

Adaptability in learners is shown when faced with new situations or challenges [10]. These abilities are essential for teamwork, as those with cognitive flexibility adjust well to new roles, diverse tasks, and various team dynamics. They excel in compromising and valuing diverse opinions in teams [64].

### Research question and hypothesis

The surveyed-above literature review illustrated that Padlet is still underutilized in higher education, specifically in healthcare professions. Moreover, the pivotal role of flexible thinking in learning has prompted much less research interest in the context of healthcare disciplines and online collaborative learning via Padlet. To address this gap, the primary objectives of this study were threefold. First, it aimed to design an intervention program (outlined below) that utilizes collaborative learning facilitated by Padlet. Secondly, it sought to evaluate how health management students perceived this intervention and its impact on their collaborative learning experiences. The third goal of the study was to evaluate the impact of students’ flexible thinking in the context of learning might influence their perception of the benefits derived from this instructional activity in the realm of online collaborative learning.

The following research question and hypothesis were examined:

- **Q1.** How health management students might perceive an intervention program facilitated by Padlet in relation to their collaborative learning experiences. Based on the literature review, which indicated a positive impact of Padlet usage on collaborative learning, it was hypothesized that the participants would acknowledge the advantages of the intervention for their collaborative learning. Furthermore, an effort was made to identify the collaborative learning constructs most significantly affected by the intervention.
- **Q2.** How might students’ flexible thinking in learning inform their different perceived collaborative learning on Padlet? Previous studies indicated the centrality of students’ flexible thinking in determining their tendency to embrace new technology-enabled learning tools (e.g., [10, 60]). Therefore, we hypothesized that students’ flexible thinking might contribute to their perception of Padlet utilization as beneficial to OCL – students who perceive themselves as more flexible would be more receptive to adopting the proposed technological tool (Padlet) and hence would tend to appreciate its function as a collaborative learning platform enabler. An effort will be made to identify the most contributive factor/s of flexible thinking in learning (learning technology acceptance; open-mindedness in learning; and adapting to new learning situations, [10] to their perceived collaborative learning performance on Padlet.

Confounding variables that might affect the research variables (e.g., age, ethnicity, grade point average [GPA], gender, and year of study) were addressed to examine and control their potential effect on the research constructs thereby allowing the examination of the associations between the research constructs over and above potential individual differences that might have existed between the students.

## Method

### Participants

Data for the analysis were gathered from 100 Israeli undergraduate students of a Health Management program (covering patient-doctor relations, quality of service in the healthcare system, and ethics and patient rights), of whom, 58% were 2^nd^-year students and 42% were 3^rd^-year students. The students’ mean age was *M* = 24.71 (*SD* = 5.85), and 88% were females. In relation to ethnicity, 56% were Jews, and 44% Arabs (Muslims). Thirty-one percent reported working in health professions (A growing percentage of students are already part of the healthcare workforce as clinicians or administrators - *Profession*). GPA was checked on a five-point scale: 1 = *50–60*; 2 = *61–70*; 3 = *71–80*; 4 = *81–90*; 5 = *91–100*. The most frequent category was 4 (51%), followed by 3 (35%). Researchers emphasized prior to obtaining consent that the questionnaires were both anonymous and voluntary. Finally, participants were assured that no identifying information about the courses would be processed. The research was approved by the college’s Ethics Committee (Certificate Number: 300).

### Measurements

#### Student perceptions of collaborative learning via Padlet

This newly designed scale [59] was used to measure students’ perceived learning performance in online collaborative learning. The measures were adapted to suit the context of learning via Padlet. The original instrument consisted of 29 items along seven factors of online collaborative tools (5 items), for example, ‘The platform has allowed me to establish personal connections with the members of my team’; collaboration with peers (4 items), for example, ‘I actively exchange my ideas with group members regarding project’; student engagement (5 items), for instance, ‘The project work has favored my personal relationships with my peers and teachers’; idea-generating (4 items), for example, ‘My groupmates are devoted to generating new ideas’; idea organizing (4 items), for example, ‘I organize knowledge with my groupmates’; intellectual convergence (5 items), for instance, ‘My groupmates and I use transfer ideas into knowledge to improve project efficiency’; and students’ learning outcome (2 items), for example, ‘Online learning, by using the platform, improves my learning performance’. In the current study, three items were added to the latter factor: ‘The learning experience with the Padlet has contributed to my ability to adapt to new learning technologies’; ‘The learning experience with the Padlet made me more open-minded to the opinions of others’; and ‘The learning experience with the Padlet made me feel able to cope with new learning situations’. A 6-point Likert-style format was used ranging from 1 = *strongly disagree* to 6 = *strongly agree*.

Exploratory factor analysis was used to determine the validity of this newly designed scale. To determine the number of factors to keep in a principal component analysis we used the statistical method of parallel analysis, also known as Horn’s parallel analysis. Based on O’Connor’s work [65], the analysis yielded a five-factor solution when comparing between the total variance explained table (eigenvalues > 1) to the random data eigenvalues, as can be learned from Table 1.

**Table 1:** Parallel analysis results

Component	Total Variance Explained	Random Data Eigenvalues
1	15.46	1.76
2	2.48	1.55
3	2.01	1.39
4	1.33	1.25
5	1.12	1.11

Based on the parallel analysis, a principal component analysis with a fixed number of five factors to be extracted (item loadings > 0.40) followed by a varimax rotation was used. All scale items were subjected to a principal component analysis. The factors accounted together for 70.0% of the variance. Six items were omitted due to low loading results (< 0.40) or when loaded on an irrelevant factor (a total of 26 items):

1. Online collaborative tools and student engagement (Tools for Collaboration and Engagement [TCE], 7 items, α = 0.94).
2. Intellectual Convergence (IC, 5 items, α = 0.81).
3. Idea-Generating and Organizing (IGO, 5 items, α = 0.88).
4. Students’ Learning Outcome (SLO, 5 items, α = 0.90)
5. Collaboration with Peers (CP, 4 items, α = 0.87).

#### Flexible thinking in learning (FTL)

This 17-item scale measures cognitive flexibility. It was designed by Barak and Levenberg [10]. It evaluates an individual’s inclination to think flexibly in contemporary learning situations. The scale consists of three factors: (1) learning technology acceptance (TA, 5 items, α = 0.89), for example, ‘I adjust quickly to new learning technologies’; (2) open-mindedness in learning (OM, 7 items, α = 0.82), for example, ‘Even when I am convinced I am right, I listen to other learner’s opinions’; and (3) adapting to new learning situations (AL, 5 items, α = 0.89), for example, ‘adjust myself to changes in learning conditions without difficulty’. A 6-point Likert-style format was used ranging from 1 = *strongly disagree* to 6 = *strongly agree*.

Exploratory factor analysis was used to determine the validity of the scale, with a fixed number of three factors to be extracted (item loadings > 0.40). All scale items were subjected to a principal component analysis followed by a varimax rotation. The factors accounted together for 64.81% of the variance. The result yielded a three-factor solution, confirming the structure of the scale, as previously suggested and confirmed by its designers.

Table 2 displays the descriptive statistics of the research sub-constructs. Following the general guidelines for skewness and kurtosis (suggesting that if the number is greater than + 1 or lower than -1, then the distribution is skewed, flat or peaked, [66], it can be learned that the distributions can be considered normal for only three sub-constructs: Open-mindedness in learning, Adapting to new learning, and Intellectual Convergence.

**Table 2:** Descriptive statistics of the research sub-constructs

*Construct*	Sub- construct	*Mean*	*Std. Deviation*	*Skewness*		*Kurtosis*	
		Statistic	Statistic	Statistic	Std. Error	Statistic	Std. Error
Flexible thinking in learning	TA	4.66	0.88	-0.97	0.24	1.90	0.48
	OM	4.88	0.59	-0.19	0.24	-0.41	0.48
	AL	4.49	0.82	-0.44	0.24	0.00	0.48
Student perceptions of collaborative learning via Padlet	TCE	4.85	1.07	-1.40	0.24	1.91	0.48
	IC	4.83	0.76	-0.62	0.24	0.41	0.48
	IGO	5.00	0.85	-1.42	0.24	2.85	0.48
	SLO	4.93	0.90	-1.39	0.24	2.87	0.48
	CP	4.88	0.90	-1.43	0.24	3.32	0.48

*TA* Learning technology acceptance, *OM* Open-mindedness in learning, *AL* Adapting to new learning situations, *TCE* Tools for Collaboration and Engagement, *IC* Intellectual Convergence, *IGO* Idea Generating and Organizing, *SLO* Students’ Learning Outcome, *CP* Collaboration with Peers

### Procedure

The program examined here is based on models of Padlet-mediated OCL. The process involves online collaborative group work aimed at creating a shared database and completing projects that draw on it [10, 59, 67]. To achieve this goal, learners must demonstrate positive interdependence, in which every individual is responsible both for their own learning and their contribution to the group [68]. The program, which emphasizes the learning process, is characterized by an active exploration led by the students’ choices, with the instructor acting as a guide but not the sole source of knowledge [69].

The program included several stages. First, the students discussed the pedagogic objective of using Padlet. They received information about the features of Padlet and understood how to post content on the platform. They also received information about what they will be expected to do, including posting content, asking questions, and offering comments.

The students studied the Padlet board to learn about its uses and options, utilizing the platform’s advantages to get to know each other and the instructor and divide into study groups. To this end, in the first stages of the course, the students freely used a Padlet board to introduce themselves in a creative way. This initial introduction served as the basis for their work with Padlet and was an important motivational tool to promote their involvement in the course, as each student was given an equal opportunity to participate [4, 6, 28].

In the second stage, the students were presented with ill-structured problems related to the course content. These problems were presented on a Padlet board (Board 1). At this point, each group had to choose one of the problems and analyze it from the three perspectives discussed in the course—healthcare provider, patient, and organization. In addition, every group was instructed to find two academic articles (that met a certain set of requirements) and present them in the chosen context on a new Padlet board (Board 2). Board 2 was organized by topics, according to the given problems, so that every group presented the information collected and its specific perspective under that category. Categories included topics such as violence in healthcare facilities, medical confidentiality, quality of health services, etc. Further, the board was divided into sub-topics, providing a platform for creating shared databases where students could post questions, comments, articles, and multimedia content according to the thematic categories [1].

The content uploaded to Board 2 was accessible to all the groups, forming a shared pool of academic knowledge. Containing numerous articles, it was classified into various categories, according to the chosen problem and course topics. At the next stage, the students chose one problem and addressed it from three perspectives—healthcare provider, patient, and organization.

Next, using the database they had created, the students presented and explained their solutions to the chosen problem from the three perspectives, employing the shared knowledge base and suggesting possible ways that the health system may address the issue. The final work was presented on a Padlet board in different formats using various technologies, per students’ preferences (e-posters, presentations, videos, and other media, using PowerPoint, ThingLink, or other software they were familiar with). This stage reflected a key advantage of Padlet, as a flexible platform that empowers each learner individually by allowing them to choose their preferred form of presentation [70].

**Fig. 1:**
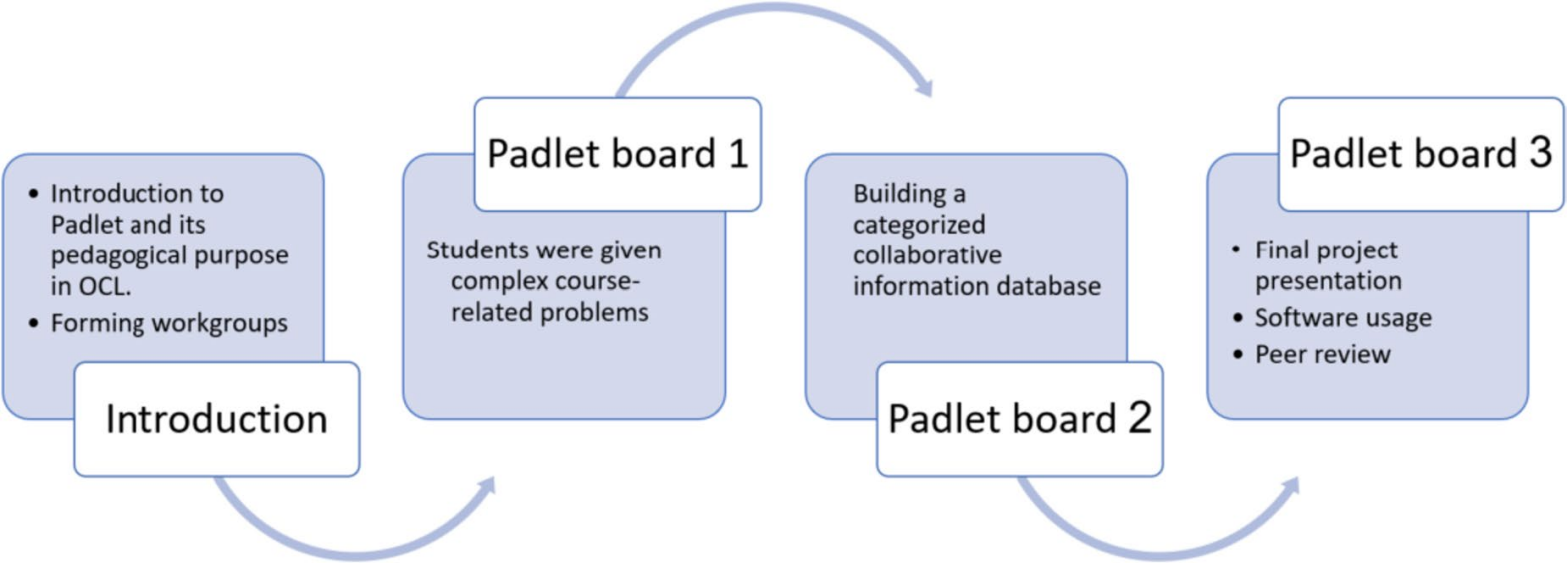
The instructional method process - the participant’s progression in the study

The products were uploaded to Padlet Board 3, where all the work produced by the students was collected. The students were required to view other participants’ work presented on the board and to respond critically by asking questions, adding information, or offering additional insights. The summary of the process is illustrated in Fig. 1.

### Data analysis

Data were analyzed by using paired samples *t*-tests and Partial Least Squares - Structural Equation Modeling (PLS-SEM) [66]) with SmartPLS 3 software. It is important to highlight that when facing scenarios where meeting the rigorous criteria of conventional multivariate methods becomes challenging, particularly those related to assumptions like normal data distribution, the utilization of Partial Least Squares Structural Equation Modeling (PLS-SEM) emerges as a favorable approach. PLS-SEM offers enhanced adaptability in comparison to covariance-based SEM (CB-SEM), primarily due to its characteristic of not imposing assumptions about the underlying data distribution. Consequently, in instances where data distribution assumptions are problematic, PLS-SEM stands out as a more suitable alternative. For the analytical process, the collected data underwent examination using PLS-SEM as outlined in the work by Hair et al. [66]. The SmartPLS 3 software was employed for conducting this analysis, harnessing the capabilities of PLS-SEM to yield valuable insights.

## Findings

**Fig. 2:**
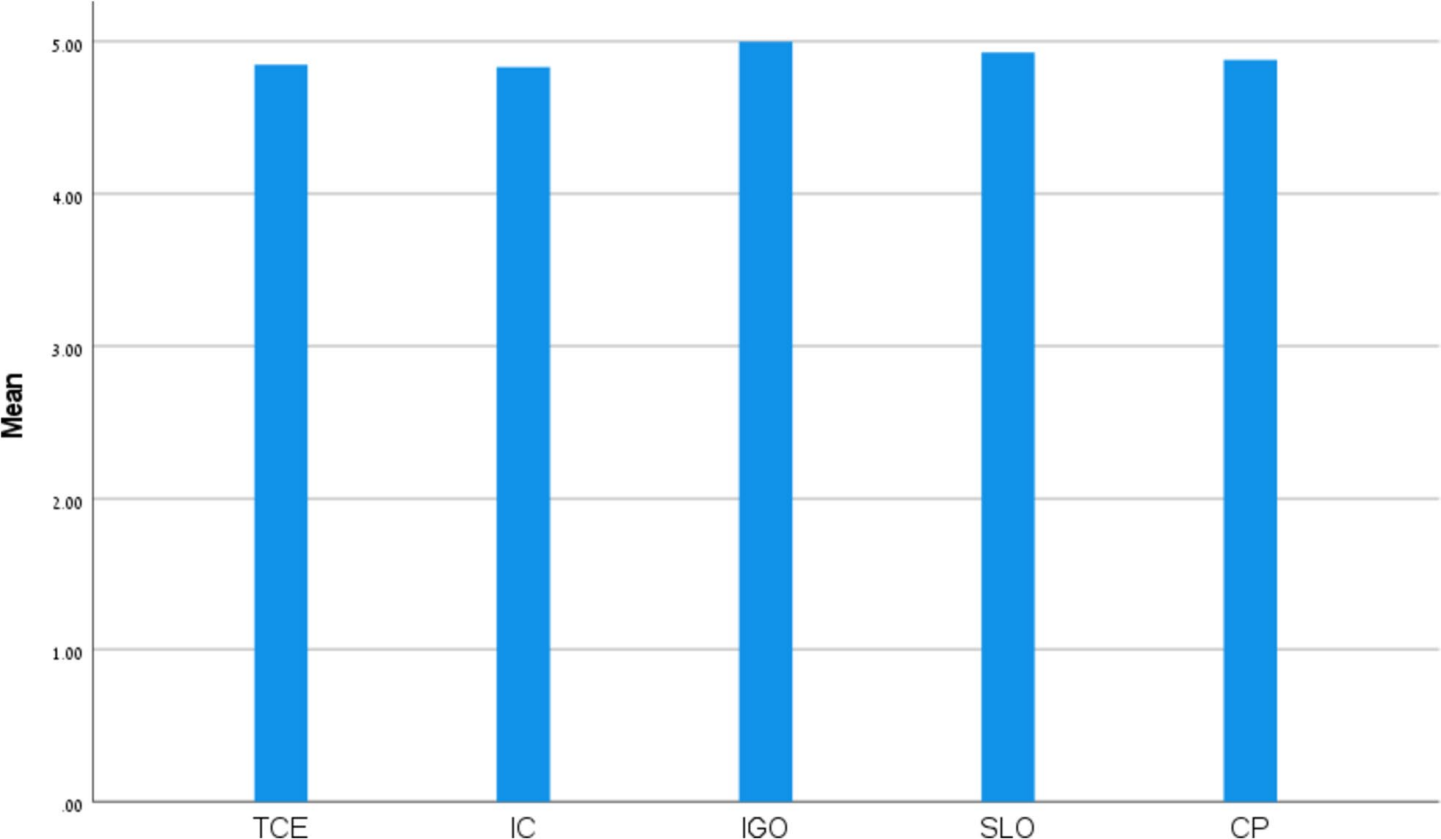
Mean results for student perceptions of collaborative learning via Padlet (sub-constructs). Note: Tools for Collaboration and Engagement (TCE); Intellectual Convergence (IC); Idea Generating and Organizing (IGO); Students’ Learning Outcome (SLO); Collaboration with Peers (CP)

Paired samples *t*-tests were used to determined the effect of the intervention on students’ perception of the collaborative learning via Padlet in relation to the following subfactors: Tools for Collaboration and Engagement (TCE); Intellectual Convergence (IC); Idea-Generating and Organizing (IGO); Students’ Learning Outcome (SLO); and Collaboration with Peers (CP). Based on Table 2, the mean results of all the subfactors can be considered high, ranging from 4.83 to 5.00. The lowest mean result was shown for Intellectual Convergence, and the highest for Idea-Generating and Organizing. According to Table 3 and Fig. 2, Idea-Generating and Organizing was found significantly higher than Tools for Collaboration and Engagement and Intellectual Convergence.

**Table 3:** Paired samples t-test results for student perceptions of collaborative learning via Padlet

Pair	*t*	*df*	*P*	*Cohen’s d*
TCE - IC	0.16	99	0.88	0.96
TCE - IGO	2.07	99	0.04	0.73
TCE - SLO	0.93	99	0.36	0.87
TCE - CP	0.47	99	0.64	0.69
IC - IGO	2.20	99	0.03	0.75
IC - SLO	1.27	99	0.21	0.75
IC - CP	0.58	99	0.57	0.82
IGO - SLO	0.83	99	0.41	0.84
IGO - CP	1.98	99	0.05	0.60
SLO - CP	0.55	99	0.58	0.89

*TCE* Tools for Collaboration and Engagement, *IC* Intellectual Convergence, *IGO* Idea Generating and Organizing, *SLO* Students’ Learning Outcome, *CP* Collaboration with Peers

**Fig. 3:**
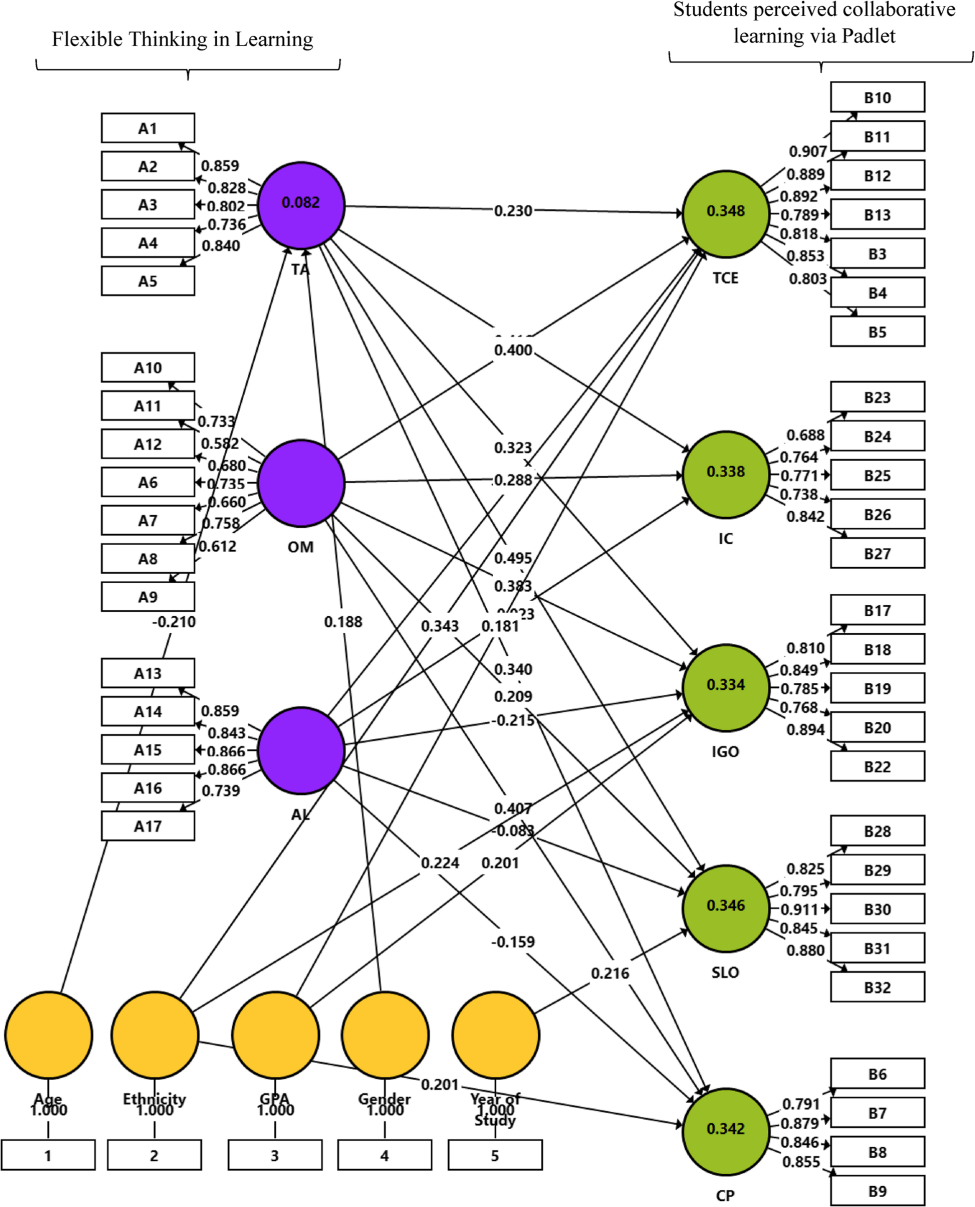
Model 1. Analysis results of research model by SmartPLS. Note: Learning technology acceptance (TA); Open-mindedness in learning (OM); Adapting to new learning situations (AL); Tools for Collaboration and Engagement (TCE); Intellectual Convergence (IC); Idea Generating and Organizing (IGO); Students’ Learning Outcome (SLO); Collaboration with Peers (CP)

Model 1 (Fig. 3) was constructed to check the second research question. It includes two main constructs of *flexible thinking in learning*, represented by three constructs on the left (Learning technology acceptance [TA]; Open-mindedness in learning [OM]; and Adapting to new learning situations [AL]); and *Student learning performance in online collaborative learning,* represented by five constructs on the right, (Tools for Collaboration and Engagement [TCE]; Intellectual Convergence [IC]; Idea-Generating and Organizing [IGO]; Students’ Learning Outcome [SLO]; and Collaboration with Peers [CP]). Additionally, the effects of all the background variables on the above constructs were measured, the results are illustrated in Table 4. Based on these results the following variables were entered into the model: age, ethnicity GPA, gender, and year of study to enable controlling their effects on the main factors. Paths were specified according to the significant results related to background variables presented in Table 4 (the results are boldfaced).

**Table 4:** Significance analysis of the direct effects, background variables included

Paths	Direct Effect	*t* value	*P* value
AGE -> AL	0.02	0.27	0.79
AGE -> CP	0.08	0.93	0.35
AGE -> IC	0.02	0.20	0.84
AGE -> IGO	0.11	1.03	0.30
AGE -> OM	0.12	1.06	0.29
AGE -> SLO	0.07	0.60	0.55
**AGE -> TA**	**-0.24**	**2.44**	**0.02**
AGE -> TCE	0.03	0.32	0.75
AL -> CP	-0.18	1.44	0.15
AL -> IC	-0.03	0.30	0.76
AL -> IGO	-0.21	1.80	0.07
AL -> SLO	-0.09	0.77	0.45
AL -> TCE	-0.15	1.25	0.21
Ethnicity -> AL	-0.01	0.05	0.96
**Ethnicity -> CP**	**0.28**	**3.13**	**0.00**
Ethnicity -> IC	0.06	0.55	0.58
**Ethnicity -> IGO**	**0.28**	**2.87**	**0.00**
Ethnicity -> OM	-0.06	0.49	0.63
Ethnicity -> SLO	0.17	1.58	0.11
Ethnicity -> TA	0.08	0.74	0.46
**Ethnicity -> TCE**	**0.35**	**4.06**	**0.00**
GPA -> AL	0.15	1.23	0.22
GPA -> CP	0.15	1.72	0.09
GPA -> IC	0.16	1.78	0.08
**GPA -> IGO**	**0.20**	**2.62**	**0.01**
GPA -> OM	0.10	0.86	0.39
GPA -> SLO	0.09	1.16	0.25
GPA -> TA	0.13	1.37	0.17
**GPA** -> **TCE**	**0.17**	**2.10**	**0.04**
Gender -> AL	0.15	1.64	0.10
Gender -> CP	-0.06	0.72	0.48
Gender -> IC	-0.10	1.17	0.24
Gender -> IGO	-0.07	1.14	0.26
Gender -> OM	-0.01	0.09	0.93
Gender -> SLO	-0.05	0.77	0.44
**Gender -> TA**	**0.20**	**2.23**	**0.03**
Gender -> TCE	-0.04	0.39	0.70
OM -> CP	0.36	2.98	0.00
OM -> IC	0.26	2.29	0.02
OM -> IGO	0.35	2.99	0.00
OM -> SLO	0.21	1.67	0.10
OM -> TCE	0.39	3.32	0.00
Profession -> AL	-0.11	0.99	0.32
Profession -> CP	0.02	0.31	0.76
Profession -> IC	0.00	0.05	0.96
Profession -> IGO	0.12	1.21	0.23
Profession -> OM	-0.02	0.13	0.90
Profession -> SLO	-0.05	0.47	0.64
Profession -> TA	-0.14	1.60	0.11
Profession -> TCE	-0.13	1.53	0.13
TA -> CP	0.37	2.31	0.02
TA -> IC	0.43	3.69	0.00
TA -> IGO	0.37	2.53	0.01
TA -> SLO	0.49	3.63	0.00
TA -> TCE	0.25	1.63	0.10
Year of study -> AL	-0.05	0.53	0.60
Year of study -> CP	-0.10	1.19	0.24
Year of study -> IC	-0.01	0.07	0.94
Year of study -> IGO	0.00	0.01	0.99
Year of study -> OM	-0.12	1.23	0.22
**Year of study -> SLO**	**0.23**	**3.03**	**0.00**
Year of study -> TA	-0.04	0.40	0.69
Year of study -> TCE	0.06	0.74	0.46

Significant results related to background variables are boldfaced

*TA* Learning technology acceptance, *OM* Open-mindedness in learning, *AL* Adapting to new learning situations, *TCE* Tools for Collaboration and Engagement, *IC* Intellectual Convergence, *IGO* Idea Generating and Organizing, *SLO* Students’ Learning Outcome, *CP* Collaboration with Peers

Table 5 displays the bootstrap routine analysis results for Model 1 (Fig. 3). It can be learned from the table that adapting to new learning situations (AL) was found non-significantly related to the dependent variables reflecting the students’ learning performance in online collaborative learning, excluding idea-generating and organizing (IGO) accompanied by a low negative coefficient result.

**Table 5:** Significance analysis of the direct effects for Model 1

Paths	Direct Effect	*t* value	*P* value
AL -> CP	-0.16	1.14	0.26
AL -> IC	-0.02	0.22	0.83
AL -> IGO	-0.22	1.70	0.09
AL -> SLO	-0.08	0.74	0.46
AL -> TCE	-0.14	1.08	0.28
Age -> TA	-0.21	2.60	0.01
Ethnicity -> CP	0.20	2.57	0.01
Ethnicity -> IGO	0.22	2.92	0.00
Ethnicity -> TCE	0.34	4.54	0.00
GPA -> IGO	0.20	2.76	0.01
GPA -> TCE	0.18	2.18	0.03
Gender -> TA	0.19	2.16	0.03
OM -> CP	0.41	3.47	0.00
OM -> IC	0.29	2.68	0.01
OM -> IGO	0.38	3.43	0.00
OM -> SLO	0.21	1.92	0.06
OM -> TCE	0.40	3.34	0.00
TA -> CP	0.34	2.22	0.03
TA -> IC	0.42	3.83	0.00
TA -> IGO	0.32	2.55	0.01
TA -> SLO	0.50	3.91	0.00
TA -> TCE	0.23	1.62	0.11
Year of Study -> SLO	0.22	3.03	0.00

*TA* Learning technology acceptance, *OM* Open-mindedness in learning, *AL* Adapting to new learning situations, *TCE* Tools for Collaboration and Engagement, *IC* Intellectual Convergence, *IGO* Idea Generating and Organizing, *SLO* Students’ Learning Outcome, *CP* Collaboration with Peers

Open-mindedness in learning [OM] positively affected the dependent variable’s sub-factors. The highest significant result was shown between this variable and collaboration with peers (CP) and tools for collaboration and engagement (TCE). Lower results were obtained between this variable and the following variables: idea-generating and organizing (IGO), intellectual convergence (IC), and students’ learning outcome (SLO).

Next, learning technology acceptance (TA) significantly affected the dependent variables, excluding tools for collaboration and engagement (TCE). The highest result was shown between this factor and students’ learning outcome (SLO), followed by intellectual convergence (IC), collaboration with peers (CP), and idea-generating and organizing (IGO).

In relation to the background variables, age has negatively affected learning technology acceptance (TA); ethnicity (Arab students) was found positively connected to collaboration with peers (CP), idea-generating and organizing (IGO), and tools for collaboration and engagement (TCE). Student GPA was positively linked to idea-generating and organizing (IGO), and tools for collaboration and engagement (TCE). Finally, students’ year of study was positively connected to their learning outcome (SLO).

### Model evaluation

Variance Inflation Factor (VIF) values were checked for collinearity. The results of all sets of predictor constructs in the structural model showed that the values of all combinations of endogenous and exogenous constructs are below the threshold of 5 [66] ranging from 1.00 to 1.65. The coefficient of determination (*R*^*2*^) values for the endogenous factors ranged from 0.33 to 0.35 these values can be considered moderate [66]. The change in the *R*^*2*^ value (*f*^*2*^ effect size) showed that the background variables had very low effect sizes, lower than 0.1 on the endogenous latent variables, excluding the ethnicity variable (*f*^*2*^ = 0.15). The highest effect size was found between learning technology acceptance (TA) and students’ learning outcome (SLO, *f*^*2*^ = 0.25), followed by intellectual convergence (IC, *f*^*2*^ = 0.25). In addition, moderate *f*^*2*^ effect size results were obtained for open-mindedness in learning [OM] and three depended variables: collaboration with peers (CP), idea-generating and organizing (IGO), and tools for collaboration and engagement (TCE), where *f*^*2*^ ranged from 0.16 to 0.18. Finally, the blindfolding procedure was used to assess the predictive relevance (*Q*^*2*^) of the path model. Values larger than 0 suggest that the model has predictive relevance for a certain endogenous construct [66]. The *Q*^*2*^ values ranged from 0.05 to 0.24.

## Discussion

This study had three objectives. Firstly, it sought to develop an intervention program that leveraged collaborative learning through Padlet. Secondly, it aimed to assess how health management students perceived this intervention and its impact on their collaborative learning experiences. Additionally, the third objective of the study aimed to investigate how students’ flexibility in their approach to learning might influence their perceptions of the benefits derived from this instructional activity within the context of online collaborative learning. The analysis has corroborated our hypothesis that students’ flexible thinking might positively contribute to their perception of Padlet utilization. According to our empirical model, in general, students who perceived themselves as more flexible were found more receptive to utilizing the proposed technological tool (Padlet) and hence tended to appreciate its function as a collaborative learning platform enabler. This can offer valuable guidance to educators looking to refine their teaching methods and improve students’ learning results. Prioritizing the use of tools for Idea-Generating and Organizing will contribute significantly to enriching the learning experience and enhancing student outcomes.

Another finding showed that open-minded students were more receptive to utilizing Padlet for collaboration with peers and perceived it as a useful tool for engagement. This finding indicates that those who tend to listen to other opinions, are open to feedback and criticism, and tend to consider various possibilities from different perspectives in the learning process, also perceived the Padlet as more sufficient in terms of enabling collaborative learning. This can be corroborated by previous studies underscoring the link between students’ inclination to think flexibly and preferences to learn in collaboration in a technologically supported learning environment [71]. However, the least effective flexible thinking sub-factor was adapting to new learning situations. This sub-factor was found to be non-significantly related to the dependent sub-factors excluding idea-generating and organizing to which it was negatively linked (with a very low coefficient result). In other words, being able to adjust to new learning situations and changes in learning processes does not necessarily contribute to student appreciation of collaborative learning via technology. It seems that the factor of adapting to new learning situations covers student tendency to adjust to new learning environments in general, but is not directly related to online technology-enabled collaborative learning, and therefore failed to significantly contribute to students’ perception of Padlet utilization as a collaborative tool [1, 2].

Lastly, learning technology acceptance significantly affected students’ perception of the Padlet as a useful technological tool, on four out of five sub-factors with an emphasis put on students’ learning outcomes. These outcomes encompass accomplishing tasks more quickly and improving learning performance, followed by intellectual convergence, collaboration with peers, and idea-generating and organizing. The Padlet platform seems to aid students in solving new problems with their peers. The participants found it useful while generating ideas from individuals and efficiently organizing them or integrating different sources and types of ideas with their group mates. Other studies also supported the idea of technological tools integration into problem-based learning and showed the benefit of these tools to student learning outcomes [46, 53, 72, 73]. This study adds to the corpus of previous work by linking the learners’ technology acceptance inclination to the way they appreciate an e-platform contribution to their problem-based learning outcomes.

In relation to the background variables, age has negatively affected learning technology acceptance. This finding is consistent with findings indicated in previous studies showing the connection between age and technology perceptions and calling for an age-sensitive design of specific technologies [74]. Ethnicity (Arab students) was found positively connected to collaboration with peers, idea-generating and organizing, and tools for collaboration and engagement. These are all sub-factors of the dependent variable of students’ perceived collaborative learning via Padlet. This finding mainly indicates that minority students appreciated the e-platform in relation to collaborative learning in problem-based learning relative to their Jewish counterparts. This corresponds to findings showing that the Jewish students’ positive attitudes toward collaborative learning have decreased after the implementation of a collaborative face-to-face activity compared to the group of Arab students. This was explained by the Arab students’ proclivity towards openness to diversity which increased their positive attitudes towards collaborative learning. Qualitative data revealed several explanations for these findings related to students’ experienced challenges during the collaborative activity [75]. The current study adds to previous work by hinting at another learning environment based on an e-platform where these cultural differences are exhibited. Finally, more experienced students in terms of year of study and those with higher GPAs tended to appreciate the Padlet platform utilization on a variety of sub-factors. This shows that individual differences might also play a role in technology acceptance [76]. It should be noted that our findings generally approved the main hypothesis above and beyond the impact of those background variables.

As indicated by this study, students may not readily embrace new technologies, especially those who do not perceive themselves as possessing flexible thinking skills. Hence, taking into account the implications for educators, they must contemplate whether and how to foster flexible thinking throughout the learning process. Researchers have emphasized the significance of exposing students to innovative learning environments and creative experiences, such as flipped classrooms and blended learning, to enhance their flexibility [77]. Others [17, 78, 79] have suggested employing diverse assessment methods, such as self and peer assessment or reflective journal writing, to promote students’ flexibility and creative thinking. We maintain that mere participation in a single learning activity using an e-platform is unlikely to lead to deep engagement. To achieve this outcome, particular attention should be directed toward designing and implementing multiple collaborative online or face-to-face experiences that encourage students to adapt to new situations, thereby facilitating flexible thinking.

### Limitations and future directions

The present work features several limitations and directions for future research. In this study, student perceptions were gauged using a self-reporting survey. According to some studies this methodology could lead to biases and strong divergence between subjective and objective assessments, therefore, data gained should be interpreted cautiously [80]. However, it is noteworthy that students’ perceptions are central to their learning [81], and may help to understand their learning experiences.

Moreover, the cross-sectional nature of the data might prevent definitive statements about causality. This will require longitudinal data gathering. It should be noted that alternate models might explain the relationships other than the one tested in this study. For example, Barak [71] measured university students’ flexible thinking according to their expertise in information and communication technology (ICT). She showed that technology-proficient students are more likely to be flexible in thought than those who are less technology-savvy. Moreover, technology-proficient students who tended toward collaborative learning were also inclined to think flexibly. Despite its limitations, one notable strength of this study is its potential to advance our comprehension of the role played by technologically supported OCL, especially with less-explored tools like Padlet, in attaining educational objectives and fostering high-order learning outcomes, as perceived by undergraduate students.

## Conclusions

Throughout this article, we have attempted to convey how students’ flexible thinking in learning might affect the way they assessed their learning performance in an online collaborative platform of Padlet. It mainly underscores the vital role that flexible thinking plays in assisting students in adapting to emerging collaborative technologies. Undoubtedly, students must be ready for a global environment, and the design of collaborative activities is pivotal in their preparation. Nevertheless, as indicated in this study, new technologies may not find ready acceptance among students, particularly those who do not perceive themselves as flexible thinkers. Hence, special attention should be paid to the design and implementation of multiple collaborative online or face-to-face usages to encourage the student to adapt to new situations thereby facilitating flexible thinking. In turn, as shown by this study, the latter might increase the probability of new platform acceptance. In today’s dynamic healthcare market, health profession students and educational institutions must adopt 21st-century learning environments. This involves cultivating open-mindedness, embracing flexibility in integrating information, processing knowledge, and making alternative decisions. Developing flexible thinkers is crucial for building a more sustainable, adaptable healthcare system. Additionally, as demonstrated in this study, flexibility motivates managers and medical professionals to embrace innovative technologies and teamwork methods. This enables them to assess arguments, explore alternative solutions to real-world issues, and adapt their approaches collaboratively when faced with new challenges.

## Data Availability

Data are available upon request.

## References

[1] Mehta KJ, Miletich I, Detyna M. Content-specific differences in Padlet perception for collaborative learning amongst undergraduate students. Res Learn Technol. 2021;29. 10.25304/rlt.v29.255.

[2] Rosnida AD, Zainor Z. Padlet as an educational tool: pedagogical considerations and lessons learnt. In: Proceedings of the 10th International Conference on Education Technology and Computers (ICETC ‘18). New York: Association for Computing Machinery; 2018. pp. 156–162. 10.1145/3290511.3290512.

[3] DeWitt D, Alias N, Siraj S. Collaborative learning: interactive debates using Padlet in a higher education institution, presented at the International Educational Technology Conference (IETC 2015), Istanbul, Turkey. Retrieved from https://core.ac.uk/reader/162014460. Accessed 10 Jan 2022.

[4] Rashid AA, Yunus MM, Wahi W (2019). Using Padlet for collaborative writing among ESL learners. Creat Educ.

[5] Rosnida AD, Zainor Z. Padlet as an educational tool: pedagogical considerations and lessons learnt. In: Proceedings of the 10th International Conference on Education Technology and Computers ICETC. Association for Computing Machinery; 2018. pp. 156–162. 10.1145/3290511.3290512.

[6] Zhi Q, Su M. Enhance collaborative learning by visualizing process of knowledge building with Padlet. In: International Conference of Educational Innovation through Technology (EITT). Wuhan: Institute of Electrical and Electronics engineers (IEEE); 2015. pp. 221–225. 10.1109/EITT.2015.54.

[7] Gasmi AA, Thomas M. Academic writing in the flipped EFL classroom: a case study on student engagement in Oman. In: Loucky JP, Ware JL, editors. Flipped instruction methods and digital technologies in the language learning classroom. IGI Global; 2017. pp. 232–251.‏ 10.4018/978-1-5225-0824-3.ch010.

[8] Hayes SC, Luoma J, Bond F, Masuda A, Lillis J (2006). Acceptance and commitment therapy: model, processes, and outcomes. Behav Res Ther.

[9] Ivanova O, Gnatyshina E, Uvarina N, Korneeva N, Savchenkov A (2021). The wheel of science: a model for managing scientific activities in higher education as a factor in developing flexible skills of the youth in the region. Think Ski Creat.

[10] Barak M, Levenberg A (2016). Flexible thinking in learning: an individual differences measure for learning in technology-enhanced environments. Comput Educ.

[11] Naamati-Schneider L. The effect of digitalization on service orientation and service perception among Israeli healthcare professionals: a qualitative study. Digit Health. 2023;9. 10.1177/20552076231191892. 10.1177/20552076231191892 PMC10392152 37533775

[12] Naamati- Schneider L (2020). Strategic management as adaptation to changes in the ecosystems of public hospitals in Israel. Isr J Health Policy Res.

[13] Dolev N, Naamati-Schneider L, Meirovich A. Making soft skills a part of the curriculum of healthcare studies. In: Firstenberg MS, Stawicki SP, editors. Medical education for the 21st century. IntechOpen; 2021. 10.5772/intechopen.98671.

[14] Al-Samarraie H, Saeed N (2018). A systematic review of cloud computing tools for collaborative learning: opportunities and challenges to the blended-learning environment. Comput Educ.

[15] Bernard JS (2015). Student engagement: a principle-based concept analysis. Int J Nurs Educ Scholarsh.

[16] Young S, Nichols H. A reflexive evaluation of technology-enhanced learning. Res Learn Technol. 2017;25. 10.25304/rlt.v25.1998.

[17] Alt D, Naamati-Schneider L (2021). Health management students’ self-regulation and digital concept mapping in online learning environments. BMC Med Educ.

[18] Bravo R, Ugartemendia L, Cubero J, Uguz C, Rodríguez AB (2018). Collaborative active learning: bioimpedance and anthropometry in higher education. Adv Physiol Educ.

[19] Ansari JAN, Khan NA (2020). Exploring the role of social media in collaborative learning the new domain of learning. Smart Learn Environ.

[20] Männistö M, Mikkonen K, Kuivila HM, Virtanen M, Kyngäs H, Kääriäinen M (2020). Digital collaborative learning in nursing education: a systematic review. Scand J Caring Sci.

[21] Malik M, Fatima G, Sarwar A (2017). E-Learning: students’ perspectives about asynchronous and synchronous resources at higher education level. Bull Educ Res.

[22] Park C, Kim DG, Cho S, Han HJ (2019). Adoption of multimedia technology for learning and gender difference. Comput Hum Behav.

[23] Yadegaridehkordi E, Shuib L, Nilashi M, Asadi S (2019). Decision to adopt online collaborative learning tools in higher education: a case of top Malaysian universities. Educ Inf Technol.

[24] Thurston A, Cockerill M, Chiang TH (2021). Assessing the differential effects of peer tutoring for tutors and tutees. Educ Sci.

[25] Harasim L. Learning theory and online technology: how new technologies are transforming learning opportunities. Routledge Press; 2012.

[26] Hrastinski S (2008). What is online learner participation? A literature review. Comput Educ.

[27] Molinillo S, Aguilar-Illescas R, Anaya-Sánchez R, Vallespín-Arán M (2018). Exploring the impacts of interactions, social presence and emotional engagement on active collaborative learning in a social web-based environment. Comput Educ.

[28] Qureshi MA, Khaskheli A, Qureshi JA, Raza SA, Yousufi SQ. Factors affecting students’ learning performance through collaborative learning and engagement. Interact Learn Environ. 2021:1–21. 10.1080/10494820.2021.1884886.

[29] Balouchi S, Samad AA (2021). No more excuses, learn English for free: factors affecting L2 learners intention to use online technology for informal English learning. Educ Inf Technol.

[30] Sukendro S, Habibi A, Khaeruddin K, Indrayana B, Syahruddin S, Makadada FA, Hakim H (2020). Using an extended Technology Acceptance Model to understand students’ use of e-learning during Covid-19: Indonesian sport science education context. Heliyon.

[31] Ornellas A, Muñoz Carril PCA (2014). methodological approach to support collaborative media creation in an e-learning higher education context. Open Learn.

[32] Breen H (2015). Assessing online collaborative discourse. Nurs Forum.

[33] DiPasquale J (2017). Wiki’d transgressions: scaffolding still necessary to support online collaborative learning. Can J Action Res.

[34] Mnkandla E, Minnaar A (2017). The use of social media in e-learning: a meta synthesis. Int Rev Res Open Distrib Learn.

[35] Blieck Y, Ooghe I, Zhu C, Depryck K, Struyven K, Pynoo B, Van Laer H (2019). Consensus among stakeholders about success factors and indicators for quality of online and blended learning in adult education: a Delphi study. Stud Contin Educ.

[36] Aldholay AH, Isaac O, Abdullah Z, Ramayah T (2018). The role of transformational leadership as a mediating variable in DeLone and McLean information system success model: the context of online learning usage in Yemen. Telemat Inform.

[37] Kuo YC, Walker AE, Schroder KEE, Belland BR (2014). Interaction, internet self-efficacy, and self-regulated learning as predictors of student satisfaction in online education courses. Internet High Educ.

[38] Sarwar B, Zulfiqar S, Aziz S, EjazChandia K (2019). Usage of social media tools for collaborative learning: the effect on learning success with the moderating role of cyberbullying. J Educ Comput Res.

[39] Shapiro AM, Sims-Knight J, O’Rielly GV, Capaldo P, Pedlow T, Gordon L, Monteiro K (2017). Clickers can promote fact retention but impede conceptual understanding: the effect of the interaction between clicker use and pedagogy on learning. Comput Educ.

[40] Senkubuge F, Modisenyane M, Bishaw T (2014). Strengthening health systems by health sector reforms. Glob Health Action.

[41] Reis S (2019). The doctor in the digital age competencies needed and a road map for their achievement. Harefuah.

[42] Reis S, Visser A, Frankel R (2013). Health information and communication technology in healthcare communication: the good, the bad, and the transformative. Patient Educ Couns.

[43] Xiong P, Zhang J, Wang X, Wu TL, Hall BJ (2017). Effects of a mixed media education intervention program on increasing knowledge, attitude, and compliance with standard precautions among nursing students: a randomized controlled trial. Am J Infect Control.

[44] Grosser J, Bientzle M, Kimmerle J (2020). A literature review on the foundations and potentials of digital teaching scenarios for interprofessional health care education. Int J Environ Res Public Health.

[45] Männistö M, Mikkonen K, Kuivila H-M, Koskinen C, Koivula M, Sjögren T, Salminen L, Saaranen T, Kyngäs H, Kääriäinen M (2020). Health and social care educators’ competence in digital collaborative learning: a cross-sectional survey. SAGE Open.

[46] Alt D, Naamati-Schneider L, Meirovich A. Future Problem-Solving Practiced During COVID-19: Implications for Health Management Students’ E-Health Literacy Identity. Front Psychol. 2022;13:829243. 10.3389/fpsyg.2022.829243. 10.3389/fpsyg.2022.829243 PMC8893171 35250771

[47] Garnham WA, Betts T (2018). The Padlet Project: transforming student engagement in Foundation Year seminars. J Learn Teach.

[48] Beltrán-Martín I. Using Padlet for collaborative learning. In: HEAD’19. 5th International Conference on Higher Education Advances. Editorial Universitat Politècnica de València; 2019. pp. 201–211.

[49] Spiro RJ, Jehng JC, Nix D, Spiro RJ (1990). Cognitive flexibility and hypertext: theory and technology for the nonlinear and multidimensional traversal of complex subject matter. Cognition, education, multimedia: exploring ideas in high technology.

[50] Tseng H, Kuo YC, Walsh EJ (2020). Exploring first-time online undergraduate and graduate students’ growth mindsets and flexible thinking and their relations to online learning engagement. Educ Technol Res Dev.

[51] Garner JK (2009). Conceptualizing the relations between executive functions and self-regulated learning. J Psychol.

[52] Boot N, Baas M, van Gaal S, Cools R, De Dreu CK (2017). Creative cognition and dopaminergic modulation of fronto-striatal networks: integrative review and research agenda. Neurosci Biobehav Rev.

[53] Alt D, Naamati-Schneider L (2021). Online argumentation-based learning aided by digital concept mapping during COVID-19: implications for health management teaching and learning. Health Educ.

[54] Brown L, Haines S, Amonoo HL, Jones C, Woods J, Huffman JC, Morris ME (2021). Sources of resilience in frontline health professionals during COVID-19. Healthcare.

[55] Harvey JF, Johnson KJ, Roloff KS, Edmondson AC (2019). From orientation to behavior: the interplay between learning orientation, open-mindedness, and psychological safety in team learning. Hum Relat.

[56] Organization for Economic Cooperation and Development (OECD) (2013). Trends shaping education.

[57] Ernawati E, Bratajaya CNA (2021). Senior nurses’ perceptions of essential soft skills for novice nurses in a private hospital in Jakarta, Indonesia: a phenomenological study. Belitung Nurs J.

[58] Crystal AB, Thomas N (2022). Core competencies for combatting crisis: fusing ethics, cultural competence, and cognitive flexibility in counseling. Couns Psychol Q.

[59] Ng PM, Chan JK, Lit KK (2022). Student learning performance in online collaborative learning. Educ Inf Technol.

[60] Barak M (2014). Closing the gap between attitudes and perceptions about ICT-enhanced learning among pre-service STEM teachers. J Sci Educ Technol.

[61] Kruglanski AW, Webster DM. Motivated closing of the mind: “seizing” and “freezing.” The motivated mind. In: Kruglanski AW, editor. The motivated mind. Routledge Press; 2018. pp. 60–103.

[62] Benade L (2019). Flexible learning spaces: inclusive by design?. N Z J Educ Stud.

[63] Salomonsson K. Flexible, adaptable, employable: ethics for a new labour market. In: Willim R, Löfgren O, editors. Magic, culture and the new economy. Routledge; 2020. pp. 117–129.

[64] Partnership for 21st Century Skills (P21). P21Framework definitions. Retrieved July 13, 2015 from: http://www.p21.org/storage/documents/P21_Framework_Definitions.pd.

[65] O’Connor BP (2000). SPSS and SAS programs for determining the number of components using parallel analysis and Velicer’s MAP test. Behav Res Methods Instrum Comput.

[66] Brody C, Davidson N. Introduction: professional development and cooperative learning. In: Brody C, Davidson N, editors. Professional development for cooperative learning: issues and approaches. SUNY; 1998. pp. 3–24.

[67] Yusuf Q, Jusoh Z, Yusuf YQ (2019). Cooperative learning strategies to enhance writing skills among second language learners. Int J Instr.

[68] Alt D, Raichel N, Naamati-Schneider L (2022). Higher education students’ reflective journal writing and lifelong learning skills: insights from an exploratory sequential study. Front Psychol.

[69] Ramachandiran CR, Mahmud MM (2019). Padlet: a technology tool for the 21st century students’ skills assessment. ICEAP.

[70] Hair Jr JF, Sarstedt M, Ringle CM, Gudergan SP. Advanced issues in partial least squares structural equation modeling. Sage Publications; 2017.‏

[71] Barak M (2018). Are digital natives open to change? Examining flexible thinking and resistance to change. Comput Educ.

[72] Hursen C (2021). The effect of problem-based learning method supported by web 2.0 tools on academic achievement and critical thinking skills in teacher education. Technol Knowl Learn.

[73] Fidan M, Tuncel M (2019). Integrating augmented reality into problem-based learning: the effects on learning achievement and attitude in physics education. Comput Educ.

[74] Hauk N, Hüffmeier J, Krumm S (2018). Ready to be a silver surfer? A meta-analysis on the relationship between chronological age and technology acceptance. Comput Hum Behav.

[75] Alt D, Raichel N (2021). Precursors of college students’ attitudes towards cross-cultural collaboration: the role of group-learning activity design and openness to diversity. J Furth High Educ.

[76] Ballejos MP, Oglesbee S, Hettema J, Sapien R (2018). An equivalence study of interview platform: does videoconference technology impact medical school acceptance rates of different groups?. Adv Health Sci Educ.

[77] Thai NTT, De Wever B, Valcke M (2020). Face-to-face, blended, flipped, or online learning environment? Impact on learning performance and student cognitions. J Comput Assist Learn.

[78] Naamati-Schneider L, Meirovich A. Student guided learning-from teaching to e-learning. Rom J Multidimens Educ. 2020;12.‏ 10.18662/rrem/12.1sup2/254.

[79] Alt D, Weinberger A, Heinrichs K, Naamati-Schneider L (2022). The role of goal orientations and learning approaches in explaining digital concept mapping utilization in problem-based learning. Curr Psychol.

[80] Bowman NA (2010). Can 1st year college students accurately report their learning and development?. Am Educ Res J.

[81] Bandura A (1997). Self-efficacy – the exercise of control.

